# Threat of COVID-19 Vaccine Hesitancy in Pakistan: The Need for Measures to Neutralize Misleading Narratives

**DOI:** 10.4269/ajtmh.20-0654

**Published:** 2020-06-22

**Authors:** Yusra Habib Khan, Tauqeer Hussain Mallhi, Nasser Hadal Alotaibi, Abdulaziz Ibrahim Alzarea, Abdullah Salah Alanazi, Nida Tanveer, Furqan Khurshid Hashmi

**Affiliations:** 1 Department of Clinical Pharmacy, College of Pharmacy, Jouf University, Sakaka, Al-Jouf, Kingdom of Saudi Arabia;; 2 Primary and Secondary Healthcare Department, Tehsil Head Quarter Hospital Jaranwala, Faisalabad, Punjab, Pakistan;; 3 University College of Pharmacy, University of the Punjab, Lahore, Pakistan

## Abstract

Immediately after declaring COVID-19 as a pandemic, numerous wild conspiracy theories sprouted through social media. Pakistan is quite vulnerable to such conspiracy narratives and has experienced failures of polio vaccination programs because of such claims. Recently, two well-known political figures raised conspiracy theories against COVID-19 vaccines in Pakistan, stating that COVID-19 is a grand illusion and a conspiracy against Muslim countries. This theory is much discussed in the local community, supporting COVID-19 vaccine hesitancy. We urge healthcare authorities in Pakistan to take necessary measures against such claims before they penetrate to the general community. Anti-vaccine movements could undermine efforts to end the COVID-19 pandemic. We believe that ethical and responsible behavior of mass media, a careful advisory from the Pakistan Electronic Media Regulatory Authority, stern measures from healthcare authorities, effective maneuvers to increase public awareness on COVID-19, vigorous analysis of information by data or communications scientists, and publication of counter opinions from health professionals against such theories will go a long way in neutralizing such misleading claims. Because Pakistan is experiencing a large burden of disease, with a sharp rise in confirmed cases, immediate action is of paramount importance to eradicate any potential barriers to a future COVID-19 vaccination program.

Vaccine hesitancy remains a substantial challenge for Pakistan amid various conspiracy theories. The failure to eradicate polio from the country is primarily attributed to such theories. Of these, alleged poor quality of vaccines, questioning of dosing recommendations, religious prohibitions (“infidel vaccine”), and rumors related to the presence of active virus in the vaccines are some leading claims obstructing the anti-polio campaign in the country.^1^ Unfortunately, a conspiracy theory against COVID-19 vaccine is currently being spread in Pakistan. Recently, a renowned political commentator and columnist in Pakistan claimed that the virus was a grand illusion to target Islamic nations, designed to allow Jews to rule the world, and to include nano-chips imbedded in the bodies of people to gain control through 5G towers.^2^ A similar theory was presented by an ex-foreign minister of Pakistan, accusing the United States of inventing the virus in the United Kingdom, with subsequent transfer to China for global spread.^3^ These theories are actively discussed in the Pakistani community through social media. In the country, where vaccine hesitancy is a prime barrier to curb vaccine-preventable diseases, such conspiracy narratives may plant seeds of resistance against upcoming COVID-19 vaccination programs.

Since a long-term lockdown is not possible for many countries due to economic turmoil, availability of vaccines may be the only way to limit persistence of the pandemic. Because of a weak healthcare system, dense population, and poor compliance with hygiene practices in Pakistan, the propensity for disease spread is high. As Pakistan has already experienced vigorous resistance against polio vaccination, any negative perception among the population toward COVID-19 vaccines would have devastating implications regarding efforts to end the pandemic. We urge the government of Pakistan to take necessary measures before anti-vaccine campaigns penetrate into the local community. In this context, we share possible measures to neutralize circulating false claims against COVID-19 vaccines.

## RESPONSIBILITIES OF MASS MEDIA

Because the volume of disparate falsehoods against COVID-19 is increasing every day, the primary responsibility lies with Pakistani media to play a sensible and professional role during the ongoing health crisis. Media should avoid any exaggerated or amplified statements triggering negative perceptions related to COVID-19 among the general community. Television channels in Pakistan should avoid airing unsupported conspiracy theories about COVID-19. The most reasonable and ethical approach would be limiting discussions on COVID-19 to healthcare professionals, rather than political or business figures. Although free speech is a fundamental right of every citizen, public harm associated with false claims must be carefully weighed. Another approach that could be useful is debates that offer opinions from researchers or healthcare professionals to counter conspiracy theories.

## ROLE OF MEDIA REGULATORY AUTHORITIES

The Pakistani mass media is dynamic and has witnessed robust growth in recent years. There are more than 30 news channels in different languages currently aired in Pakistan. The Pakistan Electronic Media Regulatory Authority (PEMRA),^4^ which works in collaboration with the Ministry of Information, regulates media activities in the country and holds power to suspend or cancel licensure of news channels. Moreover, PEMRA also issues advisories to news channels on airing ethical content and refraining from airing mis- or disinformation. Although PEMRA publishes regular reports on fake and misleading news, we did not come across any action against misleading information related to COVID-19. The Pakistan Electronic Media Regulatory Authority should issue guidelines on statements regarding the COVID-19 pandemic. Moreover, any person spreading unfounded theories without evidence should be accountable to law enforcement agencies.

## ADDRESSING RELIGIOUS ELEMENTS

Current conspiracy narratives are tied to religious beliefs. We suggest that religious elements can be addressed by involving enlightened Islamic scholars in health promotion and awareness regarding COVID-19. A similar approach was adopted by the country when the polio vaccine campaign was hindered by a conspiracy theory claiming that these vaccines were monkey- or pig-derived products, which are forbidden in Islam. Because most of the population in Pakistan regards the advice of Islamic scholars highly, the government invited scholars to educate the public on the polio vaccine, particularly in regions highly resistant to vaccination. These scholars highlighted the religious underpinning for the use of preventive medicine according to Sharia law.^5^ We believe that increased involvement of local religious authorities will facilitate appropriate COVID-19 control efforts in Pakistan.

## RESPONSE FROM HEALTH AUTHORITIES

Immunization campaigns in Pakistan are controlled by the Expanded Program on Immunization (EPI), begun in 1978 in collaboration with the WHO and UNICEF. Currently, the EPI focuses on immunizing all children against eight vaccine-preventable diseases (diphtheria, tetanus, pertussis, polio, measles, tuberculosis, hepatitis B, and haemophilus influenza type b).^6^ Because the EPI staff remain in contact with the public, their role in neutralizing misleading vaccine claims and in maximizing vaccine acceptance is of paramount importance. We suggest that the EPI should swiftly respond to any anti-vaccine campaign in the country by providing accurate information to the public.

## IMPORTANCE OF PUBLIC EDUCATION

Recently, it was documented that confidence about vaccines is directly related to public awareness of infectious diseases.^7^ A large survey on “attitudes to vaccines” involving 140,000 participants around the globe showed that countries with active public-awareness campaigns against various infectious diseases achieved very high rates of agreement on vaccine safety, effectiveness, and importance.^8^ Because conspiracy theories offer ammunition to vaccine deniers, timely intervention is of utmost importance. Health authorities in Pakistan must ensure stern measures to disseminate accurate and honest information to the public. Health authorities should make it clear that they are listening and responding to the public’s questions and concerns.

## ROLE OF RESEARCHERS AND HEALTHCARE PROFESSIONALS

Keeping in view the large circulating volume of mis- or disinformation, researchers and public health educators need to build a society that is resilient to falsehood about COVID-19, a task that will only become more vital as vaccines near. Data scientists and communications researchers have the responsibility to analyze data related to such misleading information. It is not possible to stop people from spreading ill-founded rumors. However, analysis of information sources, patterns of spread, and impacts on the general community will foster effective strategies to flatten the curve of the infodemic so that misleading information cannot spread as far and as fast.^9^ In the case of vaccines, transparent information on how vaccines are made, how they work, what they contain, how they will be tested, and their effectiveness, possible risks, and side effects will useful to ensure confidence in COVID-19 vaccines when they are available.

The government of Pakistan has not taken a hard line against misleading COVID-19 claims. We believe that putting full effort into the implementation of the aforementioned measures will go a long way to mitigating the proliferation of false claims in the country, and thereby help greatly in the control of the COVID-19 pandemic in Pakistan.
